# Editorial: Functional implications of Piwi proteins and piRNAs in stem cell maintenance and development

**DOI:** 10.3389/fcell.2025.1583955

**Published:** 2025-03-17

**Authors:** Fernanda Loureiro Almeida O’Sullivan, Ani V. Das, Patricia Rojas-Ríos

**Affiliations:** ^1^ Embrapa Pesca e Aquicultura, Palmas, Tocantins, Brazil; ^2^ Rajiv Gandhi Centre for Biotechnology, Thiruvananthapuram, Kerala, India; ^3^ Departamento de Genética, Facultad de Biología, Universidad de Sevilla, Sevilla, Spain

**Keywords:** PIWI proteins, piRNAs, stem cells, development, diseases

## Introduction

PIWI proteins and their associated non-coding PIWI-interacting RNAs (piRNAs) constitute a highly conserved silencing pathway that is particularly active in animal gonads, where they play crucial roles in genome regulation. piRNAs are small non-coding RNAs, 24 to 31 nucleotides in length. PIWI proteins are loaded with piRNAs forming ribonucleoprotein complexes that recognize target mRNAs through sequence complementarity, leading to their degradation through the endonucleolytic activity of PIWI proteins. The primary function of this pathway is to suppress transposable elements (TEs) in both somatic and germline gonadal cells, thereby safeguarding genomic stability by preventing TE mobilization.

Beyond transposon silencing, emerging studies have highlighted additional roles of PIWI proteins and piRNAs in post-transcriptional gene regulation particularly in stem cell biology and development. While these functions are fundamental to germline maintenance and development, they have also been identified in somatic tissues and certain types of cancers. The continuous exploration of the PIWI-piRNA pathway has provided insights into novel regulatory mechanisms that contribute genome integrity, cell fate determination, and metabolic control in stem cells.

This Research Topic compiles the recent advancements in understanding the role of PIWI proteins and piRNAs in stem cell biology, with a particular emphasis on their regulation of gene expression at the post-transcriptional level under both physiological and pathological conditions, as well as during developmental processes. Additionally, this Research Topic includes original contributions on the role of the piRNA pathway in germline development across both vertebrates and invertebrates.

## PIWI proteins and piRNAs in stem cells and development

The *Drosophila* ovary has been a key model in uncovering the piRNA pathway, providing fundamental insights into its role in transposable element (TE) regulation. Studies in this system have demonstrated how PIWI proteins and piRNAs collaborate to suppress TE activity, ensuring genome stability in the germline. Furthermore, it has also been instrumental in discovering novel functions of the piRNA pathway in stem cell biology, particularly in gene expression regulation at multiple levels, including chromatin modifications and mRNA regulation. Claro-Linares and Rojas-Ríos provide a minireview about the diverse functional roles of PIWI proteins and their associated piRNAs in stem cell biology, with a focus on their gene regulatory mechanisms that induce or repress the translation of specific mRNAs in germline stem cells (GSCs) of *Drosophila melanogaster.* Notably, they highlight recent findings identifying piRNAs as key regulators of glycolytic mRNAs in these stem cells (Rojas-Ríos et al., 2024). Additionally, they summarize research data on the role of PIWI proteins in somatic stem cells, mainly in highly regenerative species like planarians and *Drosophila* intestinal stem cells. In a more specific original research article, Adashev et al. show that the RNA helicase Vasa, an essential factor in the piRNA pathway, is required for the survival and proliferation of male GSCs. Moreover, their data support that Vasa-mediated regulation of piRNA biogenesis ensures the proper suppression of *Stellate* elements, underscoring the importance of piRNA-guided gene silencing in germline maintenance during *Drosophila* spermatogenesis. In vertebrates, PIWI proteins are also crucial for germ cell survival and differentiation. Research in Atlantic salmon by Almeida et al. has revealed that PiwiL1, one of the vertebrate homologs of PIWI, is indispensable for germline development. The loss of the PiwiL1 N domain leads to germ cell apoptosis, suggesting a protective role in germ cell survival. Additionally, PIWI-piRNA interactions regulate the expression of genes involved in germline stemness and differentiation, thereby ensuring the continuity of the reproductive lineage. This highlights a conserved function of PIWI proteins in sustaining the germline across species.

Deregulation of PIWI proteins has been linked to multiple types of diseases including cancer. The review by Patel et al. provides a general overview of the piRNA pathway under normal conditions and its mechanisms of action. Additionally, it explores the existing literature on the emerging roles of both piRNA-dependent and independent functions of PIWI proteins in the Soma, particularly in tumor development, as well as cardiovascular and neurodegenerative diseases. Moreover, García-Silva et al., in a perspective article, discuss in detail the role of the PIWI protein PIWIL4 in acute myeloid leukemia (AML), an aggressive malignancy driven by a population of leukemia stem cells. They critically analyze recent findings on PIWIL4 in AML *in vivo*, particularly its regulatory actions at genomic level. The authors also present original expression data of PIWIL4 in blood samples from AML patients, reinforcing previous reports by Bamezai et al. (2023) that indicate PIWIL4 overexpression in this disease. Based on these findings, they propose that further investigation of the piRNA pathway in AML could contribute to the development of novel biomarkers and therapeutic strategies for improved treatment outcomes.

## Conclusion

The articles published in this topic Research Topic focus on the functional roles of the piRNA pathway in controlling the self-renewal and differentiation properties of germline and somatic stem cells, as well as during physiological and pathological development, such as cancer. Understanding these molecular processes offers valuable insights into cellular protection mechanisms and underscores the importance of maintaining PIWI/piRNA function. Therefore, this Research Topic on “Functional Implications of Piwi Proteins and piRNAs in Stem Cell Maintenance and Development” brings together cutting-edge research that deepens our understanding of these mechanisms and their broader implications in both basic biology and clinical applications.
